# Evaluation of Evaporation Fluxes for Pesticides and Low Volatile Hazardous Materials Based on Evaporation Kinetics of Net Liquids

**DOI:** 10.1021/acsomega.4c01405

**Published:** 2024-04-12

**Authors:** Olena A. Spaska, Michal Daszykowski, Yuriy G. Bushuev

**Affiliations:** Institute of Chemistry, University of Silesia in Katowice, 9 Szkolna Street, 40-006 Katowice, Poland

## Abstract

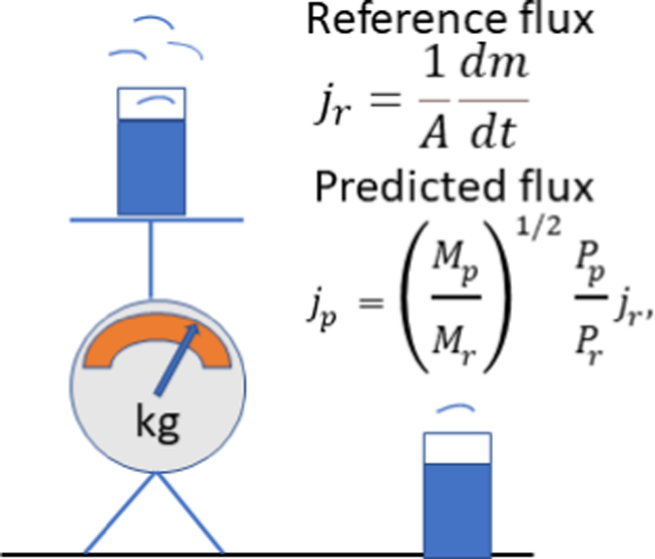

Evaporation is the phase transition process that plays a significant role in many spheres of life and science. Volatilization of hazardous materials, pesticides, petroleum spills, etc., impacts the environment and biosphere. Predicting evaporation fluxes under specific environmental conditions is challenging from theoretical and empirical points of view. A new practical method for estimating fluxes is proposed based on our experimental results and previously published data. It is demonstrated that some parameters in theoretical equations for near-equilibrium evaporation can be estimated from experiments, and these formulas can be exploited to predict steady-state evaporation fluxes in the air in a range of 8 orders of magnitude based on a single experiment carried out for nontoxic volatile compounds.

## Introduction

Evaporation is a liquid–gas phase transfer process that commonly occurs in nature. It has multiple applications in industry and is vital for environmental science. The evaporation of crude oil and petroleum products in industrial storage tanks has not only a large economic significance^1^ but also contaminates the environment.^2^ Air and soil pollution by herbicides impacts human health.^3^ Using hazardous compounds in agriculture, chemical plants, or laboratories and even dwellings creates risks to the health and safety of people and animals. For example, one of the critical parameters is the volatilization time of solid or liquid pesticides from crops, plants, and soil.^4,5^ These are challenges for environmental science, stimulating the development of methods for predicting the time scale of evaporative loss from chemical spills or estimating the concentrations of hazardous materials in the air.

The molecular mechanisms of evaporation have still not been elucidated in most cases. Theoretical investigations of fluid kinetics are limited due to the complexity of the tasks.^6^ The two most popular theories of evaporation are Hertz–Knudsen (HK)^7,8^ and statistical rate theory (SRT).^9,10^ However, their application is restricted by near-equilibrium and low-vapor-pressure cases. Under nonequilibrium conditions, evaporation and condensation are coupled. Evaporation depends on the properties of the liquid surface, while condensation depends on the liquid’s and the vapor’s properties.^11^ As a result, the evaporation and condensation coefficients used in the HK theory are different and extremely hard to measure.^8^ The coefficients depend on liquid and vapor temperatures, heat flux, and interface geometry.^12^

The SRT provides an alternative expression for the evaporation flux and does not contain fitting parameters. For some systems, calculated fluxes correspond to measured values.^8^ The theory has been used to solve a reverse task. Using experimental fluxes, vapor pressures above the interface were calculated and compared with experimental ones.^11^ However, the calculations can be done for particular experimental conditions, and compounds’ thermodynamic and spectroscopic properties (vibrational frequencies) must be known.

Generally, the evaporation flux depends on actual temperatures above and below the liquid–vapor interface, vapor pressure, and interface contamination. Even the size and shape of liquid samples affect the evaporation. The theories of millimeter or micron-sized drop evaporation and evaporation from thin films are discussed in review articles.^13,14^ Atomistic computer simulations are possible only for nanoscale systems, whose properties significantly differ from macro systems.^9,15,16^

For many practical applications and environmental science, predicting evaporation rates in the air from planar surfaces of large areas under different conditions, including airflow or wind, is necessary. Neglecting the high accuracy, based on accurately determined thermodynamic properties of the liquid–vapor systems measured for pure liquids, we have attempted to exploit the functional form of statistical theory equations to fit experimentally obtained evaporation fluxes, substituting unknown parameters with empirical coefficients depending on the environment. The present work aims to establish the relationship between evaporation fluxes and properties of volatile compounds, which hold in different environmental conditions, and proposes a method for predicting evaporation fluxes based on minimal experimental measurements. For this purpose, we measured evaporation fluxes from planar surfaces of several liquids using a weight loss method at two temperatures with and without airflow. The obtained correlations are applied to previously published^4,17^ experimental data to test the method and generalize the results.

## Methods and Materials

In environmental science, studying the evaporation of liquids or volatile solids is based on measuring their weight loss over time under controlled conditions.^18^ Evaporation may occur from different substrates, solutions,^3^ or open planar surfaces of materials under investigation.^19^

According to ASTM method D3539-87, a known volume of liquid is added to a known area of filter paper placed in a thin-film evaporometer, a cabinet with a balance. The dried air is passed through the cabinet, and evaporation kinetics is measured from 10 to 90% of the mass loss.^5,20^ However, evaporation from thin liquid films depends on the thickness of the film, wettability, porosity, texture, and other properties of the material from which evaporation occurs.^14,21−23^ Micro- and nanosized films are formed near the container walls or fibers of the material.^24^ It is challenging to maintain the film to have the same thickness during an experiment when a significant amount of liquid evaporates. The rate of molecular diffusion through a porous substrate affects the evaporation of highly volatile liquids. It was experimentally demonstrated^3^ that properties of substrates, such as treated soil, plant foliage, solid surfaces of glass or plastic, and water, influence the evaporation flux.

Another method is based on evaporation from an open planar surface of liquids when the influence of substrate properties, a meniscus, and thin films is minimal. A Petri dish is an example of a vessel for evaporation experiments, where all samples are under the same conditions. The dishes were used to study the evaporation of crude oil and multicomponent fuel mixtures.^18,19,25^ For example, the height of the dish above the oil varied from 2 to 20 mm depending on the depth of the fill.^19^ Components of such mixtures have different volatility, molecular mass, and diffusion coefficients. Diffusion-controlled concentration gradients in the liquid phase near the interface and a time-dependent decrease in more volatile compound concentrations in the mixture are expected for steady-state evaporation. Using shallow Petri dishes decreases the influence of these factors.

Steady-state evaporation intends to have nonequilibrium states in both phases, and experimental conditions can affect vapor and liquid. The weak correlation between evaporation rates and wind velocity was demonstrated for crude oil and petrol products.^19,25^ Pure hydrocarbons show different responses to the wind. For example, it was shown that the evaporation rate of heptane sufficiently increases with wind velocity, indicating boundary-layer regulation of the evaporation. At the same time, the wind weakly affects the evaporation rates of heavy hydrocarbons.^25^

Crude oil and petrol products are multicomponent mixtures of compounds with a large difference in volatility. Stirring of mixtures eliminates concentration gradients near the liquid–vapor interface. It is effective, especially in the case of thick layers of these liquids. However, stirring can disturb the shape of the interface and create air turbulence. In the present work, the evaporation of only net liquids was investigated. Thus, there was no concentration gradient in a liquid. The temperature gradients in both phases and the interfacial temperature discontinuity^8,12^ were effectively accounted for in empirical parameters.

Rahimi and Ward studied water evaporation from partially filled capillaries closed at the bottom.^11^ They demonstrated that the evaporation flux is strongly dependent on the water level in the capillary. The fastest evaporation rate was observed for the most filled capillary. However, vapor pressure and evaporation flux depend on interface curvature and thus from the diameter of the capillary.

The present work used beakers instead of Petri dishes or capillaries. The role of a meniscus is negligible in that case. A level of poured liquid is easily regulated, and its distance from a lip can be the same as in the case of a Petri dish. The advantage of beakers is that the volume of liquid in a vessel can be exploited as an additional factor controlling the evaporation flux for net liquids.

Graduated 100 mL glass beakers (SIMAX, tall form) with dimensions 48 mm (*D*) × 80 mm (*H*), conforming to standard ISO 3819, were loaded with a measured amount of liquids. We used an electronic balance from Radwag, model WTB-210, with a scale interval of 1 × 10^–6^ kg, repeatability of ±1 × 10^–6^ kg, and maximum error of ±2 × 10^–6^ kg, which is capable of measuring the evaporation rate by the weight loss method^19^ for low volatile liquids.

### Two Experimental Procedures Were Employed

1. Passive evaporation. The measurements were carried out in a room (volume ca. 60 m^3^) at 293 K and atmospheric pressure with windless conditions. The room was not ventilated and was empty during experiments, except for an experimentalist. 50 mL of liquid was poured into each 100 mL beaker. Thus, the possible influence of turbulent air currents, impacting the evaporation rates of the liquids, was minimized.
2. Evaporation under air flux. A dynamic climatic chamber (MK 240, Binder), where the temperature was kept constant (298 K), was used as the second location. Each beaker contained 75 mL of a liquid. Internal ventilators produced laminar airflow inside the chamber. This airflow generated turbulence above liquids due to interactions with beaker walls, but the turbulence was the same for all beakers in the chamber. The liquid and vapor properties, diffusion through a stagnant layer, and turbulent dispersion influence the evaporation rate.^18^ Turbulence affects the thickness of the layer and a vapor pressure gradient. The impact of turbulence depends on the phase interface position in the beaker. The liquid level can be exploited as an additional factor that controls evaporation.

Different time intervals were selected for data acquisition due to very different evaporation rates (Er). The fast experiments (2 min) were performed for acetone and *n*-pentane in the climatic chamber. It prevented the cooling of the liquids. For *n*-hexadecane, measurements took more than 100 h for passive evaporation.

Steady-state evaporation rates (Er) were calculated by linear fitting weight loss (Δ*m*) with time (*t*), as presented in Figure S1 for *n*-pentane. The strong dependence of Er on the volume of liquid in the beaker was observed (Figures S1 and S2) due to different distances from the liquid–vapor interface to the lip of the beaker. The same effect was observed for experiments performed according to the second procedure. Thus, in contrast to Petri dishes, the volume of poured liquid is an additional factor controlling the thickness of the stagnant layer and evaporation rate.

During experiments, the levels of liquids in beakers changed. Still, we controlled the linearity of kinetic curves. As presented in Figure S1a–c, even for highly volatile pentane, a statistical measure to qualify the linear regression *R*^2^ is larger than 0.999 in all cases. A special test was performed to examine the reproducibility and calculate statistical errors. For this purpose, the same volume of selected liquid was poured into six beakers, and evaporation rates were measured simultaneously under the same conditions. The kinetics of evaporation are presented in Figure S2b. For *n*-heptane, the standard deviation did not exceed 0.2 mg min^–1^ for three liquid levels in the beaker. The relative error is less than 5% for volumes used in our experimental setups.

By definition, the evaporation flux (*j*) was calculated according to the formula *j* = Er/*A*, where *A* is the liquid–vapor interface area. Fluxes were measured for the following liquids: *n*-pentane (99%), *n*-hexane (99%), *n*-heptane (99%), *n*-decane (99%), *n*-dodecane (95%), *n*-hexadecane (99%), distilled water, ethanol (96%), and acetone (99%). For simplicity, we will miss the prefix “*n-*” for hydrocarbons in the following sections.

## Theoretical Considerations

Several empirical approaches were proposed to establish a correlation between evaporation rates and the physical properties of volatile compounds and their vapors.^3,5,18^ Experimental results were summarized, analyzed, and presented as the database,^17^ which includes evaporation fluxes obtained by the ASTM D3539-87 evaporation rate test^20^ for 51 compounds covering a broad range of fluxes from 5.04 × 10^–4^ kg m^–2^ s^–1^ (acetone) to 1.27 × 10^–11^ kg m^–2^ s^–1^ (pp′-DDT).

The following considerations with regard to independent parameters used for the correlation of evaporation fluxes (*j*), measured as the steady-state rate from the unit area (*A*), correlate with *M* × *P*_s_, where *M* is the molecular mass and *P*_s_ is the vapor saturation pressure. Assuming that vapor immediately achieves *P*_s_ near the liquid surface and applying the ideal gas law, Mackay and van Wesenbeek^17^ obtained the following relation
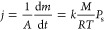
1where *k* is the empirical mass-transfer coefficient, *R* is the gas constant, and *T* is the temperature. The coefficient *k* can depend on the temperature and molecular mass. Generally, when a liquid evaporates in the air, the net flux is a sum of evaporation and condensation fluxes. The last one depends on the properties of liquid and environmental conditions. Airflow and the position of the liquid–vapor interface in the vessel (Figures S1d and S2b) affect molecular transport resistance and therefore the evaporation and condensation fluxes.

Other parameters for the correlation of fluxes were used in the article of Woodrow et al.^3^ For evaporation from the soil, they consider the linear regression equation
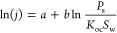
2where *a* and *b* are the adjustable parameters, *K*_oc_ is the soil adsorption coefficient, and *S*_w_ is the water solubility.

In the case of noninteractive surfaces, such as plants, glass, or plastic, they demonstrated^3^ that the modified Knudsen equation^18^ is applicable to explain the evaporation of pure compounds
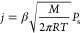
3where β is the constant. The obtained results supported the noninteractive nature of plant surfaces for freshly applied pesticides.^3^

According to the Hertz–Knudsen–Schrage (HKS) equation,^8,12^ the net evaporation flux is an algebraic sum of evaporation and condensation fluxes
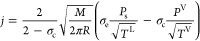
4where, in addition to the previous notations, *P*^V^ is the vapor pressure near the interface, *T*^L^ and *T*^V^ are the liquid and vapor temperatures, σ_e_ and σ_c_ are the evaporation and the condensation coefficients, respectively. The coefficients are the mean probabilities that a molecule is being emitted or captured by the liquid surface. They depend on temperature.^26^Equation 4 is based on the Maxwell–Boltzmann velocity distribution function, which calculates the number of the so-called “hot” molecules in a liquid or vapor.

A similar equation was obtained in the statistical rate theory (SRT)^12^ after introducing a few simplifications
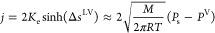
5where *K*_e_ is the equilibrium molecular exchange rate, Δ*s*^LV^ is the entropy difference between vapor and liquid. Equations 4 and 5 apply to the near-equilibrium state. Analytical calculations of fluxes based on theoretical approaches can be performed only for special cases.^11^ Most systems far from equilibrium or steady-state systems are too complex. It was shown experimentally for actual systems that *T*^L^ and *T*^V^ temperatures depend on the distance from the liquid–vapor interface.^12^ Generally, the vapor pressure *P*^V^ and its gradient above the interface are difficult to predict due to air convection or airflow, but these conditions are usual in practice.

Comparing eqs 1–5, one can mark different flux dependences on molecular mass and temperature. The flux is proportional to (*M*/*T*) × *P*_s_, *P*_s_, or (*M*/*T*)^1/2^ × *P*_s_. These equations will predict different fluxes for compounds with different molecular weights at the environment’s temperature range. The additional term from condensation flux, which depends on vapor pressure near the liquid–vapor interface, is presented in eqs 4 and 5. Neglecting possible differences in temperature for liquid and vapor and their dependence on the distance to the liquid–vapor interface,^12^ assuming *T*^V^ = *T*^L^ = *T*, eq 4 can be rewritten as
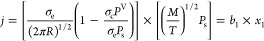
6We especially select the function’s argument *x*_1_ in such form for grouping the property of a compound (*M*), the equilibrium property of liquid–vapor coexistence (*P*_s_), and environmental temperature (*T*). Only the value of the saturated pressure may be unknown for some compounds. Still, in most cases, it can be taken from available NIST or Dortmund databases, for example, or calculated according to theoretical relations.^4,27,28^

A thin-film model of evaporation in the air was proposed previously.^29^ For simplicity, the volume below and above the liquid–vapor interface is subdivided into several layers: the stagnant liquid film, the equilibrium vapor (Knudsen), air–diffusive, and turbulently mixed air layers. Molecule transport through these layers defines the evaporation flux. In the case of diffusion-limited evaporation, resistance is significant in a field far from the Knudsen layer.^30^ The partition coefficient *K* = *C*_air_/*C*_liq_ = (*PM*)/(*RTρ*), where ρ is the density of the liquid, is an indicator of the controlling phase. It was shown that if *K* ≪ 10^–3^, the air controls an evaporation flux.^31^ One of our tasks is to evaluate fluxes for very low volatile compounds, for which *P* is extremely low; thus, diffusion through the air layer controls the flux.

Equation 6 has three additional parameters compared with those of eqs 1 and 3. We made several assumptions.

(1) eq 6, proposed to near-equilibrium conditions, can be exploited for steady-state evaporation in the air even when air circulation near the interface is significant and affects the thickness of the air-diffusive layer. Air convection, airflow, and turbulence make the diffusion layer thinner and accelerate evaporation.
(2) The factor governing evaporation at different conditions is σ_c_*P*^V^/σ_e_*P*_s_. There is a discussion in the literature about the values of evaporation and condensation coefficients.^8^ These parameters are absent in the SRT formula, eq 5. We assume the coefficients are equal (σ_e_ = σ_c_) for simplicity.
(3) We hypothesize that only the *P*^V^/*P*_s_ ratio or, in other words, only the deviation of vapor pressure in the air-diffusive layer from saturation pressure controls the steady-state evaporation flux, and this ratio is a constant for all liquids taken at the same environmental conditions.

Thus, we generalized the equations and assumed their applicability to steady-state evaporation from planar liquid surfaces. For a closed vessel, evaporation and condensation fluxes are equal, *P*^V^ = *P*_s_, and the net flux *j* = 0. Thus, all poorly defined factors affecting evaporation are contained in one empirical parameter (*b*_1_), and eq 6 corresponds to eq 3. However, we are going to show that *b*_1_ weakly depends on compounds and strongly on environmental conditions. In other words, *b*_1_ = const if the measurements were performed under the same conditions. If the assumptions are appropriate, then experimental fluxes are proportional to (*M*/*T*)^1/2^ × *P*_s_.

## Results and Discussion

**Figure 1:**
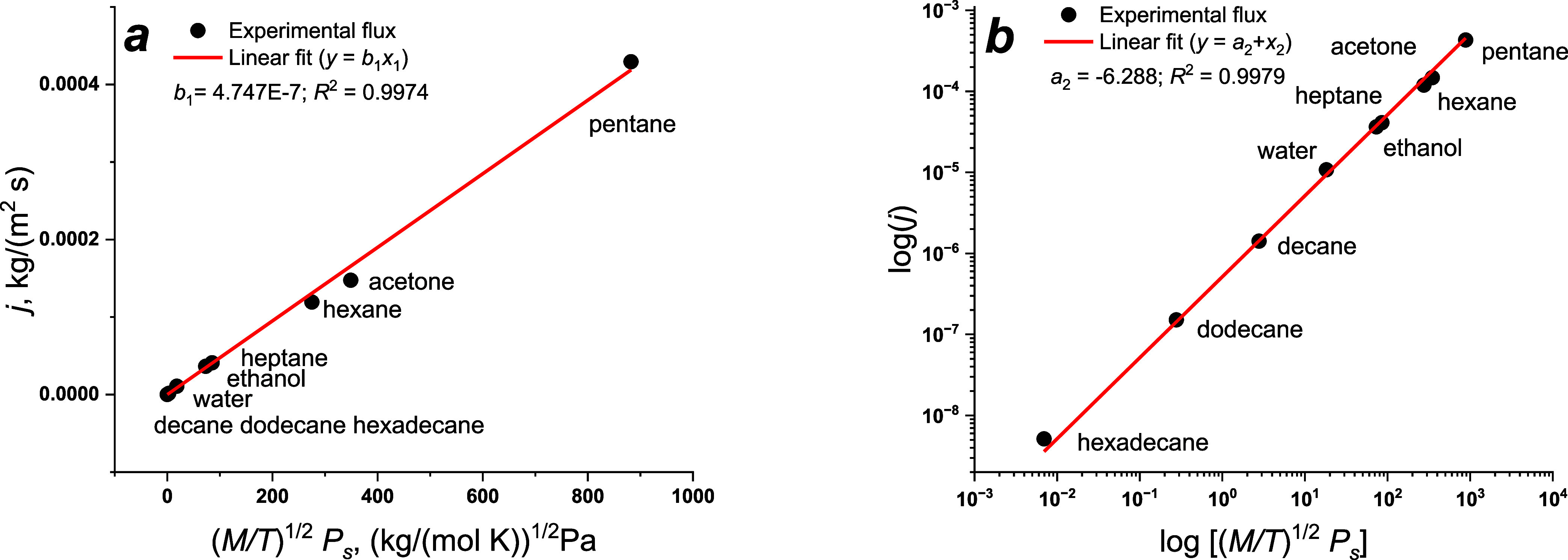
Experimental evaporation fluxes from planar liquid surfaces measured at 293 K and windless conditions (procedure 1) are linearly fitted according to eq 6 (a) or eq 7 (b).

Our experimental data were plotted and linearly fitted according to eq 6. The results are presented in Figure 1a. If evaporation fluxes differ by several orders of magnitude, then the coefficient of determination *R*^2^ mostly depends on highly volatile liquids.

Equation 6 can be rewritten in the logarithmic form to include all liquids in correlation
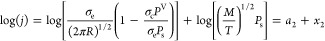
7where *a*_2_ is the constant and *x*_2_ is the function’s argument. The results of data fitting on the log–log scale are shown in Figure 1b. For both approximations, the *R*^2^ value is larger than 0.997. For comparison, the linear fitting according to eq 1, presented in Figure S3, demonstrates a worse correlation, *R*^2^ = 0.9897. Absolute percentage errors of flux predictions are shown in Figure S4. The accuracy of the prediction is higher if one uses eq 7. The maximal deviations from experimental values are ca. 60 and 30% for eqs 1 and 7, respectively.

Thus, only one adjustable parameter is needed to fit experimental data, which differ by 5 orders of magnitude. The analysis of our results confirms our assumptions and simplifications. The coefficient *a*_2_ = log(*b*_1_) is the same for all liquids under consideration at the same experimental conditions. Here, the evaporation fluxes of only nine liquids were measured. Additional tests are needed to prove our assumptions.

**Figure 2:**
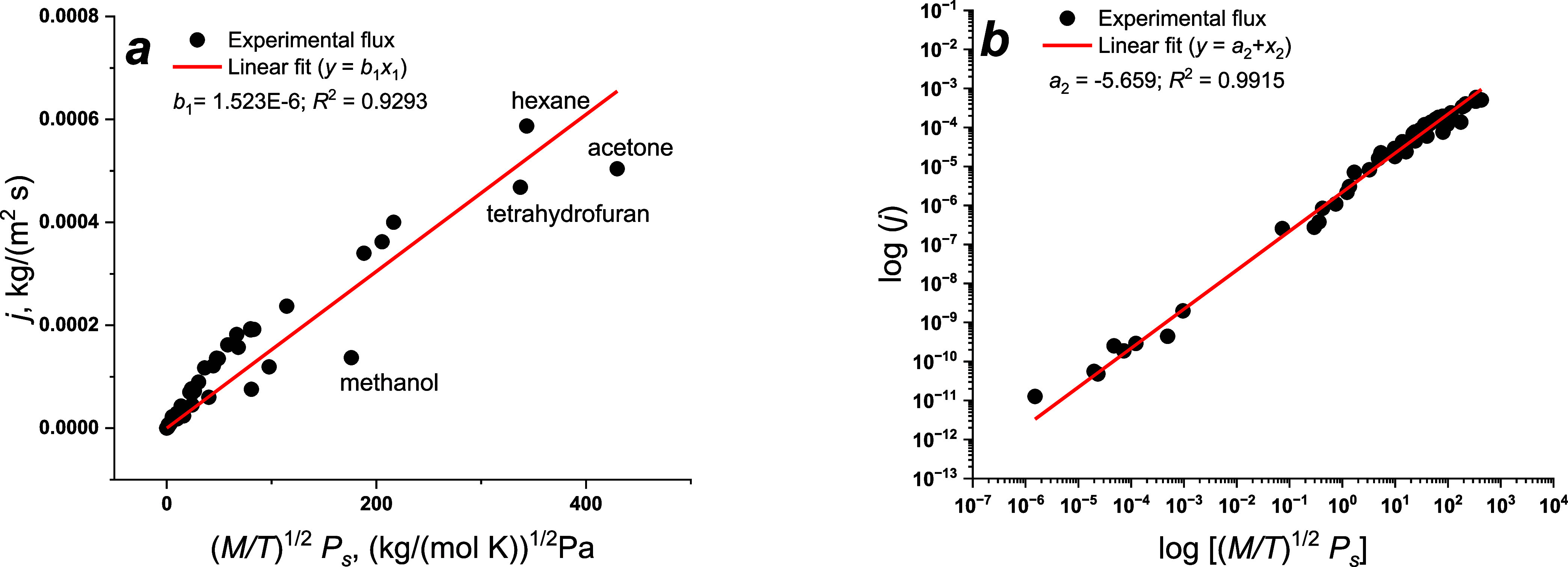
Experimental fluxes from filter paper measured at 298 K and fitted according to eq 6 (a) or eq 7 (b).

For the first test, we took “as is” previously collected and published^17^ evaporation fluxes for 51 compounds measured at 298 K according to the ASTM D3539 test.^20^ These experimental conditions and the method differ from those used in our investigation. The results of experimental data fitting according to eqs 6 and 7 are shown in Figure 2. The analyzed data were collected from several publications, and we suppose that the experimental conditions could differ.

Meanwhile, Figure 2b demonstrates a good correlation in a range of 8 orders of magnitude due to the log–log scale. This scale is recommended to exploit for crude estimation of evaporation fluxes for extremely low volatile compounds. In the case of well-controlled experiments and close volatilities of a reference system and a compound for which evaporation is predicted, it is better to use eq 6. Linear correlations of evaporation fluxes with the selected arguments testify to the validity of our assumptions and hypothesis.

In the second test, we performed experiments in the climatic chamber at 298 K and using a constant airflow speed (procedure 2). The rotation frequency of the ventilators in the chamber regulates the airflow speed. It was the same for all samples. Each beaker contained 75 mL of liquids instead of 50 mL in the previous experiments. Thus, three main experimental conditions accelerating the evaporation rates were changed. The experiments took 2–4 min for highly volatile liquids, whereas, in the case of hexadecane, only 6 mg was evaporated after 400 min.

**Figure 3:**
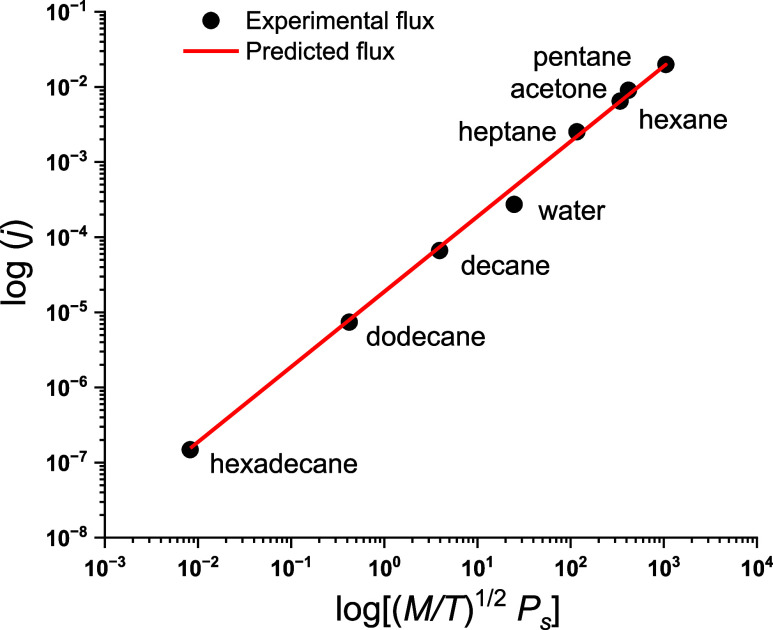
Experimental and predicted fluxes for different liquids from planar surfaces at 298 K and constant airflow. Measurements were performed in a climatic chamber (procedure 2). *j*, [kg/(m^2^ s)]; (*M*/*T*^1/2^) *P*_s_, [kg/(mol K)]^1/2^ × Pa.

We attempted to predict fluxes for a set of selected liquids using the measured evaporation flux for pentane. According to eqs 6 and 7, the coefficient *b*_1_ = *j*/*x*_1_ = exp(*a*_2_). It was calculated and applied to all liquids under consideration. The experimental data and the corresponding predictions are shown in Figure 3. Thus, we demonstrated that evaluating evaporation fluxes in a range of 5 orders of magnitude is possible using only one measurement. Even in the extreme case of the pentane–hexadecane pair, the predicted value of 1.55 × 10^–7^ kg/(s m^2^) slightly exceeds the experimental one (1.48 × 10^–7^ kg/(s m^2^)). The largest deviation is observed for water. One of the reasons for this difference is the humidity of air, which can affect the *P*^V^/*P*_s_ ratio in some circumstances. Several nontoxic volatile compounds may be selected for the experiment to increase the accuracy of evaluation.

**Figure 4:**
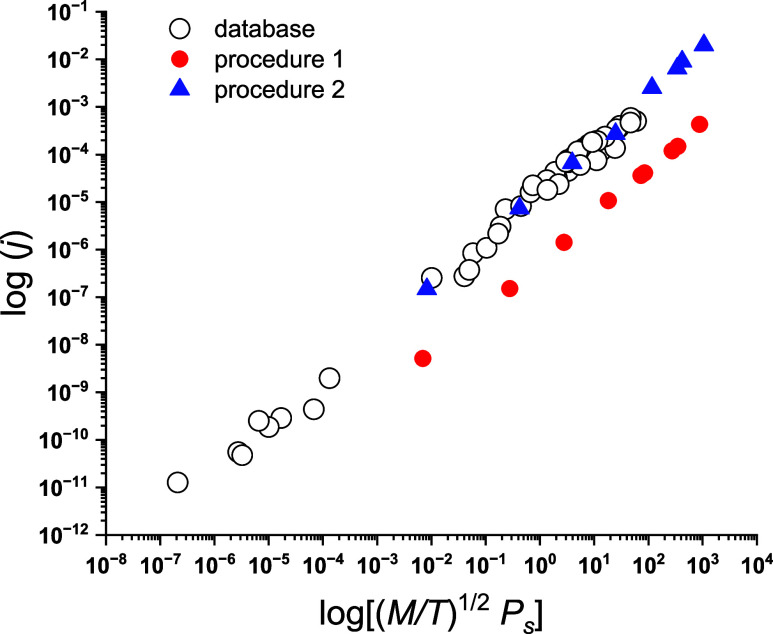
Experimental evaporation fluxes were obtained by using different methods and under different environmental conditions. *j*, [kg/(m^2^ s)]; (*M*/*T*^1/2^) *P*_s_, [kg/(mol K)]^1/2^ × Pa.

All experimental data under consideration are presented in Figure 4 as a log–log plot. According to eq 7, the slopes of fitting lines are the same and the difference is only in the *a*_2_ value. Accidentally, two sets of data, obtained from database^17^ and experimental procedure 2, are close to each other, *a*_2_ = −5.66 and −4.75, respectively. Thus, two different methods provide similar results. The temperature and airflow used for measurements with the standard evaporimeter can be tuned to correspond to the actual environmental conditions. Suppose weight loss for a reference liquid evaporated from leaves of plants, crops, or soil is the same as that in the evaporimeter. In that case, experiments can be performed using the standard method to increase the accuracy of the evaluated fluxes for hazardous materials.

Based on our results, a new method of evaporation rate evaluation for actual environmental conditions can be proposed. For this purpose, the conditions for the liquids selected for the experimental measurements must be the same. According to eqs 6 or 7, only one adjustable parameter (*b*_1_ or *a*_2_) is needed to estimate other compounds’ evaporation flux and rate. Thus, experiments can be performed instead of measuring the rates for hazardous or low-volatility materials, for example, for nontoxic compounds with high volatility. It may be any reference liquid, even water, but in this case, the air humidity will affect the accuracy of the prediction. Only the molecular mass and saturation pressure are needed for the calculations. If the reference liquid and volatile compounds, those evaporation fluxes will be predicted, are at the same environmental conditions, then
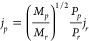
8where the indexes *p* and *r* denote predicted and reference properties, respectively.

For illustration, we evaluated fluxes for some low volatile hazardous compounds using *a*_2_ = −4.752, the value (eq 7) obtained from experiments performed according to procedure 2. Experimental and evaluated fluxes for two pesticides are 1.27 × 10^–11^ and 2.69 × 10^–^11 for pp′-DDT and 5.53 × 10^–11^ and 3.51 × 10^–11^ kg/(m^2^s) for toxaphene, respectively. Considering the extrapolation within 7 orders of magnitude, these results show good agreement.

**Figure 5:**
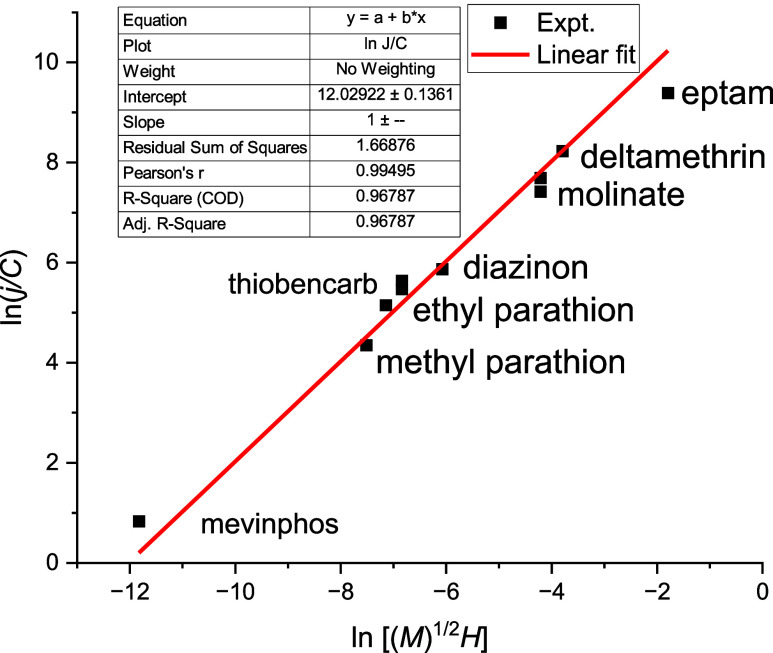
Correlation of concentration normalized pesticide emission rates from aqueous solutions (*j*/*C*) with properties of the compounds. *j*, [μg/(m^2^ h)]; *C*, [mg/L]; (*M*^1/2^) *H*, [(kg/mol)^1/2^ × Pa × L/mg].

The final test of our assumptions was performed for pesticides applied to water. Previously published experimental data^4^ taken “as is” were analyzed for this purpose. They are presented in Table S1 and Figure 5. The pressure *P*_s_ in eq 6 has to be substituted by the partial pressure of the compound *P* that depends on the concentration in the aqueous solution, *C*.^4,18^ For ideal solutions, *P* = *HC*, where *H* is the Henry volatility, which is defined as *H = P*_s_/*S*_w_, where *S*_w_ is the compound solubility in water. Thus, eq 6 can be rewritten and applied to aqueous solutions
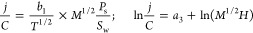
9

All presented data were linearly fitted at the same temperature with one adjustable parameter *a*_3_. The results of the fitting are shown in Figure 5. The *R*^2^ > 0.96 testifies to the quality of approximation. Parameters *a*_3_ and *b*_1_ are approximately constants under the same environmental conditions for all compounds under consideration. To estimate evaporation fluxes, one can measure the flux only for one liquid and calculate the adjustable parameter. Statistics can be collected to improve the accuracy of predictions. Thus, the proposed method can be applied to pure compounds and solutions.

## Conclusions

The generalized statistical physics equations were used to calculate evaporation fluxes for the near-equilibrium states. Based on our experiments and published data, we demonstrated that only one adjustable parameter is enough to estimate the evaporation fluxes of compounds over a broad range, covering several orders of magnitude. This parameter strongly depends on environmental conditions and weakly on the material’s properties. The reference and evaluated pure compounds’ molar masses and saturation pressures must be known for the estimation. For dissolved compounds, one must know solubility and Henry volatility. A simple method for evaporation flux measurements has been proposed. The evaporation rates of liquids poured in beakers can be regulated not only by the temperature and airflow speed but also by their volumes. Measurements performed under actual environmental conditions for reference liquids can be supplemented by results obtained in a laboratory for a larger set of compounds. It will increase the accuracy of the predictions. Nontoxic, highly volatile liquids can be the reference systems.

## Supporting Information Available

Kinetics of pentane evaporation at 293 K, no wind conditions, for three levels of liquid in the beaker; rate of evaporation vs volume of pentane in a 100 mL beaker; kinetics of heptane evaporation at 293 K, with no wind conditions, measured for six beakers; rate of evaporation vs volume of heptane; experimental evaporation fluxes from planar liquid surfaces measured at 293 K and windless conditions; absolute percentage errors of flux prediction; Henry’s law ratio, derived from measured vapor pressure (*P*_s_) and emission rates (*j*/*C*) for pesticides applied to water (PDF)
