# Supplementary material for: Evaluation of Evaporation Fluxes for Pesticides and Low Volatile Hazardous Materials Based on Evaporation Kinetics of Net Liquids
Source: ACS Omega. 2024 Apr 12;9(16):18617–23. doi: 10.1021/acsomega.4c01405 (PMC11044173; doi:10.1021/acsomega.4c01405)
Supplement: Supplementary file 1 — ao4c01405_si_001.pdf [file ao4c01405_si_001.pdf]

Supporting Information

Evaluation of evaporation fluxes for pesticides and low volatile hazardous materials based on evaporation kinetics of net liquids

Olena A. Spaska, Michal Daszykowski, and Yuriy G. Bushuev,*

Institute of Chemistry, University of Silesia in Katowice,

9 Szkolna Street, 40-006 Katowice, Poland.

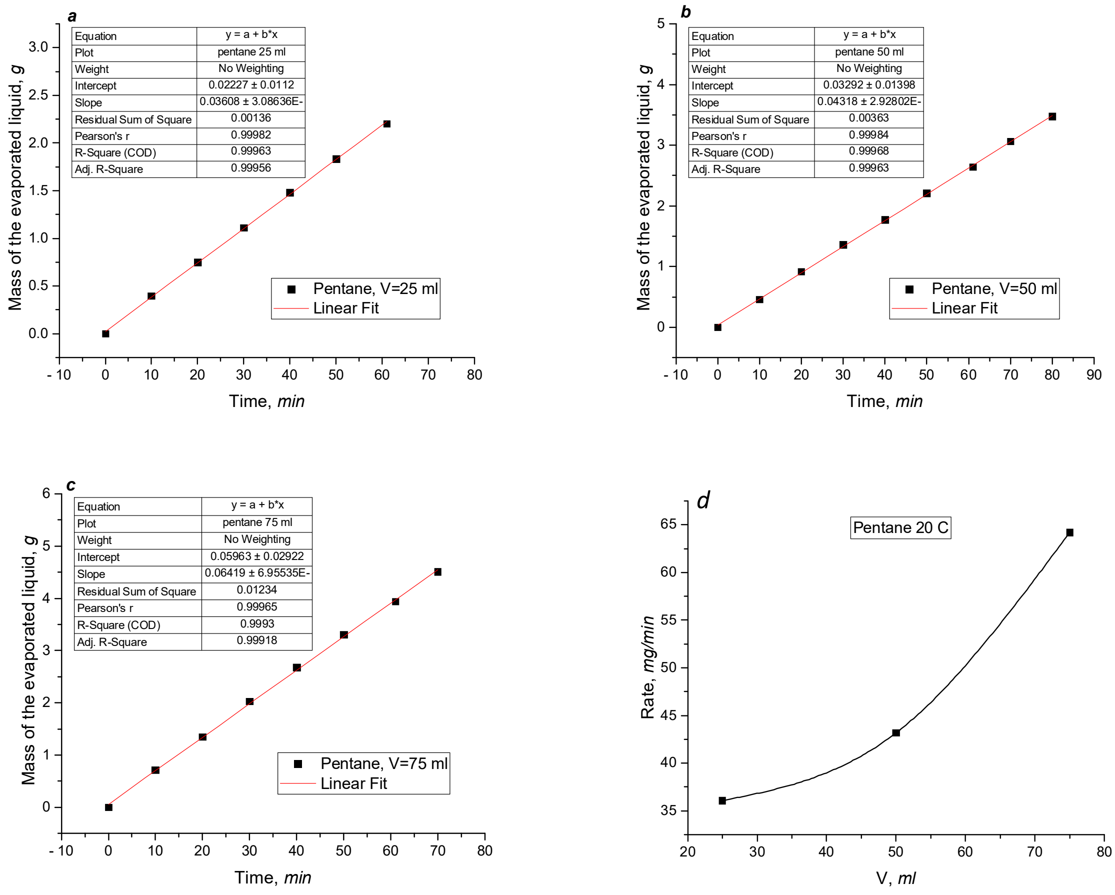

Figure S1. (*a-c*) Kinetics of pentane evaporation at 293 K, no wind conditions, for three levels of liquid in the beaker. (*d*) Rate of evaporation vs. volume of pentane in 100 ml beaker.

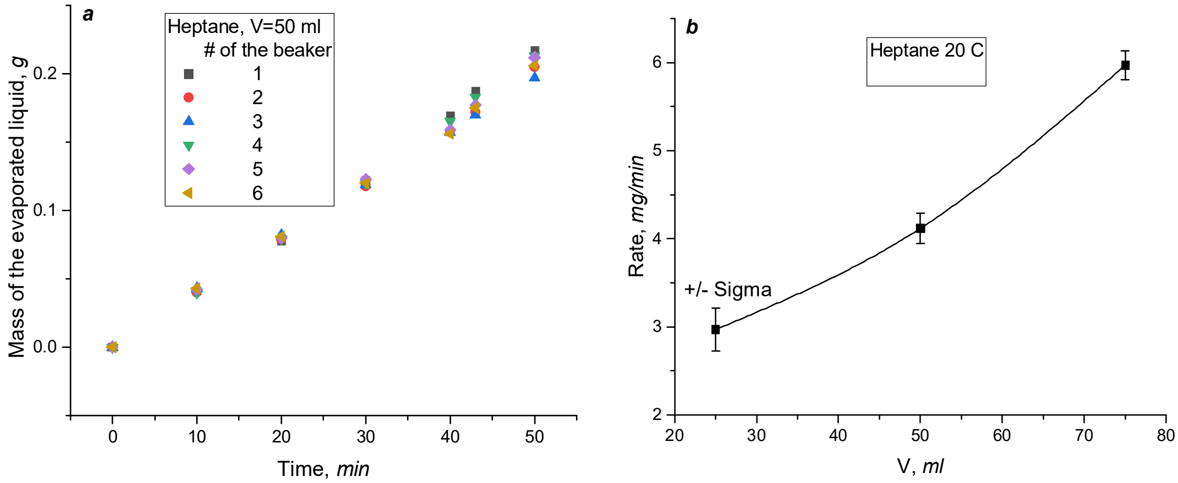

Figure S2. (*a*) The kinetics of heptane evaporation at 293 K, with no wind conditions, were measured for six beakers containing 50 ml of liquid in each beaker. (*b*) Rate of evaporation vs. volume of heptane in 100 ml beaker.

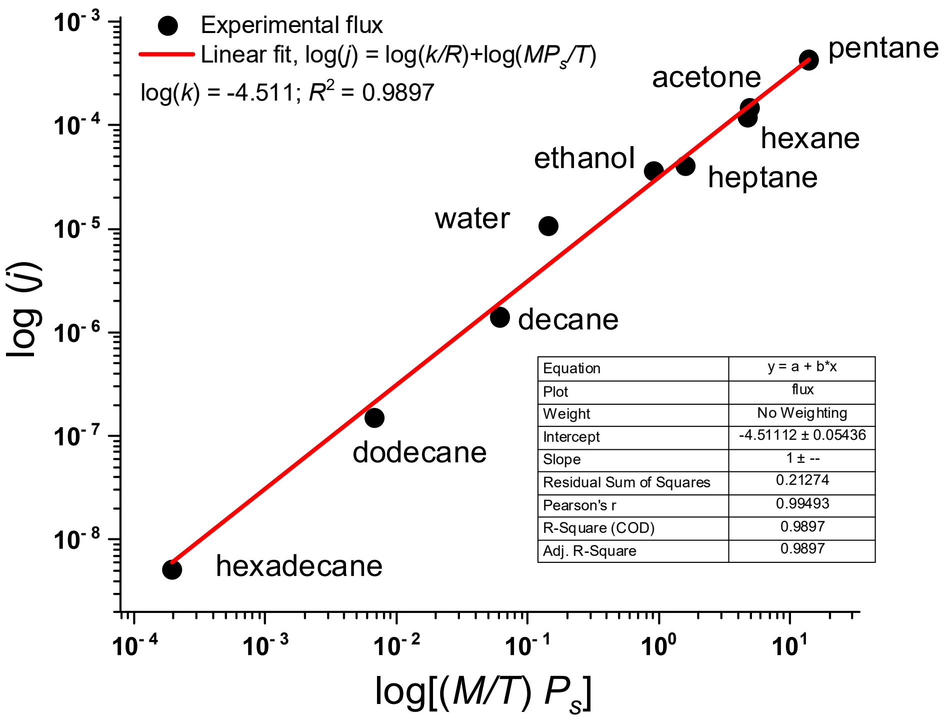

Figure S3. Experimental evaporation fluxes from planar liquid surfaces measured at 293 K and windless conditions (procedure 1) and linearly fitted according to Eq 1. j, [kg/m^2 s)]; $(M/T)\ P_s$, [(kg Pa)/(mol K)]

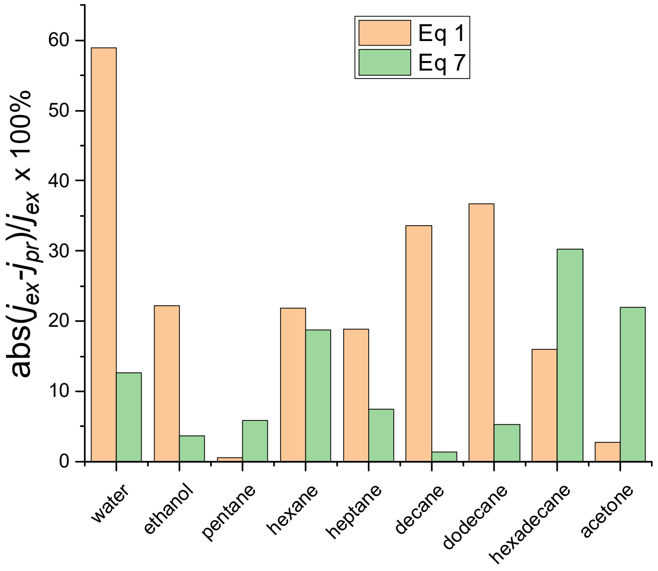

Figure S4. Absolute percentage errors of flux prediction according to Eq 1 and Eq 7.

Table S1 Henry's law ratio (Ps/Sw), derived from measured vapor pressure (Ps), and emission rates (j/C) for pesticides applied to water[1]

Compound	Sw	Ps	ln(Ps/Sw)	ln(j/C)	M
deltamethrin	0.002	$2\ 10^{-6}$	-6.908	8.23	505.206
diazinon	48.6	0.0064	-8.935	5.87	304.35
eptam	375	4.53	-4.416	9.39	189.318
ethyl parathion	15	$6.93\ 10^{-4}$	-9.982	5.15	291.26
methyl parathion	25	$8.4\ 10^{-4}$	-10.301	4.35	263.2
mevinphos	$6\ 10^{5}$	0.293	-14.532	0.83	224.149
molinate	688	0.746	-6.827	7.42	187.3
molinate	688	0.746	-6.827	7.69	187.3
thiobencarb	30	0.002	-9.616	5.64	257.78
thiobencarb	30	0.002	-9.616	5.47	257.78

Sw is the water solubility, [mg/L]. Ps is the pressure, [Pa]. j/C, [(μg/m^2h)/(mg/L)]. M is the molecular mass, [kg/mol].

(1) Woodrow, J. E.; Seiber, J. N.; Dary, C. Predicting Pesticide Emissions and Downwind Concentrations Using Correlations with Estimated Vapor Pressures. *J. Agric. Food Chem.* **2001**, *49* (8), 3841–3846. https://doi.org/10.1021/jf010358u.
